# The Study of *Escherichia coli* as Antimicrobial‐Resistant Sentinel Microorganism Isolated in the Farms of Three Districts of Ankara by MALDI‐TOF MS and Genomic Analysis

**DOI:** 10.1002/vms3.70209

**Published:** 2025-04-02

**Authors:** Didem Karademir, Banu Kaskatepe, Hilal Basak Erol, Suleyman Yalcin, Yasemin Numanoglu Cevik

**Affiliations:** ^1^ Department of Pharmaceutical Microbiology Ankara University Graduate School of Health Science Ankara Turkey; ^2^ Department of Pharmaceutical Microbiology Ankara University Faculty of Pharmacy Ankara Turkey; ^3^ Department of Microbiology Reference Laboratory and Biological Product MoH General Directorate of Public Health Ankara Turkey

**Keywords:** antibiogram, antibiotic resistance, *Escherichia coli*, extended‐spectrum beta lactamase, MALDI‐TOF MS, resistance gene, One Health

## Abstract

One Health is a unified approach that aims to sustain and maintain the health of humans, animals and the ecosystem. The environment plays an important role in the spread of resistance genes, as it is an unlimited source of antimicrobial resistance genes. *Escherichia coli* can acquire and spread resistance genes from its environment. Extended‐spectrum beta‐lactamase (ESBL)‐producing *E. coli* is a global concern because it can hydrolyse many beta‐lactam antibiotics. In this study, the aim was to isolate *E. coli* from faeces and soil samples collected from cattle, sheep and poultry in three districts (Golbası, Haymana and Cubuk) where livestock (cattle, sheep and poultry) farming activities are intensively carried out. In addition, the antibiotic susceptibility of the isolated *E. coli* was to be determined using phenotypic and genotypic methods and the presence of ESBLs was to be determined using a double‐disc synergy test. All 120 *E. coli* isolates were confirmed by the MALDI‐TOF MS method. The resistance rates of all isolates were as follows: ampicillin, 12.5%; trimethoprim/sulfamethoxazole, 6.6%; cefazolin, 0.83%; ciprofloxacin, 2.5%; ceftazidime, 0.83%; cefotaxime, 1.6% and ceftriaxone, 1.6%. Cefazolin (99.1%) and trimethoprim/sulfamethoxazole (0.83%) were determined to have intermediate susceptibility. Only one *E. coli* strain was found to be ESBL positive via phenotypic methods, and whole‐genome analysis was performed on this strain. As a result of whole‐genome analysis, ESBL‐related *CTX‐M‐14* and *TEM‐1* genes were found in the plasmids. This is the first study on the determination of antibiotic susceptibility and the presence of ESBL in *E. coli* isolated from the soil and faeces samples of farms in these regions. More studies are needed to determine and understand antibiotic resistance and ESBL positivity in environmental samples. Therefore, the One Health approach should be emphasised.

## Graphical abstract

The antibiotic susceptibility of *Escherichia coli* isolates isolated from faeces and soil samples collected from farms in three districts where livestock farming activities are intensively carried out via phenotypic and genotypic methods and the presence of extended‐spectrum beta‐lactamases (ESBLs) via a double‐disc synergy test. Only one *E. coli* strain was found to be ESBL positive via phenotypic methods, and whole‐genome analysis was performed on this strain.

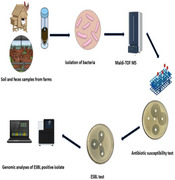

## 1 Introduction

Antimicrobial resistance causes treatment failure and long hospitalisations for serious diseases, as well as increases health care costs (Dadgostar 2019). It has become a global public health challenge of the 21st century. The European Antimicrobial Resistance Surveillance Network (EARS‐NET) and the Central Asian and Eastern European Antimicrobial Resistance Surveillance Network (CAESAR), which are epidemiological surveillance networks in Europe and Asia, have stated that antibiotic resistance has become more common in recent years (Christaki, Marcou, and Tofarides 2020).

Antimicrobial resistance can be inherent or acquired resistance. Acquired resistance is a greater concern. Microorganisms, mostly commensal, such as *Escherichia coli* and *Staphylococcus aureus*, are closely associated with community‐ and hospital‐acquired infections and can acquire antibiotic resistance genes through resistance plasmids and other mobile genetic elements (transposons and integrons). Resistance may also develop as a result of mutation or transfer of resistance genes in susceptible bacteria to other bacteria via transformation, transduction and conjugation (Abushaheen et al. 2020; Tenover 2006; Wright and Sutherland 2007).

Antimicrobial resistance can spread among humans, animals and the environment in different ways. (Ahmad, Malak, and Abulreesh 2021). Soil is considered a reservoir for resistance genes, as most antibiotics produced are derived from intrinsically resistant soil microorganisms, and organic fertilisers used in agriculture and water contaminated with faecal microorganisms cause the spread of resistant bacteria in the soil (Prestinaci, Pezzotti, and Pantosti 2015). Resistance can be transmitted to humans through direct contact with animals, and in 2007, the risk of carrying gentamicin‐resistant *E. coli* in poultry workers was 32 times greater than that in other members of the public. In addition, contact with animal products or the consumption of animal products may lead to exposure to resistant bacteria (Marshall and Levy 2011). In one study, *E. coli* O157:H7 and *Salmonella spp*. were detected in manure, compost, soil and water samples (Ahmad, Malak, and Abulreesh 2021).

Microorganisms affect the health of plants, soil, animals and humans. Microorganisms are overwhelmingly present in soil and can be considered the basis of a One Health approach (Banerjee and van der Heijden 2023). One Health approach is needed to increase scientific data through surveillance studies to address the global challenge of antimicrobial resistance. In its 2014 report, the WHO emphasised that antibiotic resistance should be addressed with a One Health approach at the local, national, regional and global levels (WHO 2014).

Extended‐spectrum beta‐lactamase (ESBL)‐producing *E. coli* is a problem in both developed and developing countries, and foods obtained from animals, especially poultry, constitute a source for humans (Collignon and McEwen 2019). ESBL‐producing Enterobacterales bacteria have genes that confer resistance to other antimicrobial agents, including fluoroquinolones and aminoglycosides, and therefore may be multidrug resistant (McDonald et al. 2021). Phenotypic or genotypic tests are performed to detect ESBLs in microbiology laboratories. Phenotypic methods are nonmolecular tests used to detect the ability of ESBL enzymes to hydrolyse different cephalosporins and are often used in clinical diagnostic laboratories because they are easy to perform and inexpensive. Genotypic tests detect the genes (*bla*_TEM_, *bla*_CTX‐M_, *bla*_OXA_ and *bla*_SHV_) responsible for the production of ESBL enzymes via molecular techniques (Falagas and Karageorgopoulos 2009; Pitout and Laupland 2008). Multidrug‐resistant *E. coli* isolates from the environment have also become widespread worldwide in recent years. So far, a large number of TEM, SHV and CTX‐M ESBL β‐lactamase enzyme variants and plasmid‐mediated AmpC β‐lactamases (pAmpC) have been identified. Among these, *bla*_KPC_, *bla*_NDM‐1_ and *bla*_OXA‐48_ are new types that have been isolated from humans and animals around the world (Igbinosa et al. 2023; Liu, Thungrat, and Boothe 2016).

Matrix‐assisted laser desorption/ionisation time‐of‐flight mass spectrometry (MALDI‐TOF MS) is a method for rapid identification of bacterial species. The method is based on the direct measurement of the ribosomal protein fingerprint of microbes, enabling high‐precision search and sensitive identification (Barreiro et al. 2017; Bizzini and Greub 2010). MALDI‐TOF MS accurately discriminates the most closely related species. However, it sometimes fails to discriminate between related species due to similar intrinsic properties. For example, it cannot distinguish *E. coli* from *Shigella* (Rychert 2019).

The aim of this study was to determine the antimicrobial susceptibilities of *E. coli* isolates from stool and soil samples collected from 39 farms (sheep, cattle and poultry farms) in three districts with intensive animal husbandry activities via phenotypic and genotypic methods and the presence of ESBLs and to determine the resistance genes of ESBL‐positive isolates by whole‐genome analysis.

## 2 Materials and Methods

### 2.1 Isolation and Identification of *E. coli* Isolates

*E. coli* isolates were collected from stool and soil samples from 39 farms (including 9 sheep, 14 cattle and 15 poultry farms) in three districts with intensive livestock farming. Soil samples were taken approximately 5 cm below the surface from inside and in front of the barns and chicken coops. A total of 184 samples were taken, 43 of which were faecal samples and 141 of which were soil samples. The samples brought to the laboratory were placed in Tryptic Soy Broth (TSB) medium and incubated at 37°C for 24 h. Bacterial samples grown in TSB medium were inoculated into MacConkey agar (MCA) and incubated overnight. In this way, lactose‐positive bacteria were inoculated on Eosin Methylene Blue (EMB) agar and incubated overnight, and the colonies with a metallic shine were evaluated as *E. coli*.

As a result of the biochemical tests, bacteria that were lactose, indole, and, methyl red positive, Voges–Proskauer and citrate negative, and bacteria that turned the medium into yellow colour in the Triple Sugar Iron (TSI) test were considered *E. coli*.

### 2.2 Identification of *E. coli* by MALDI‐TOF MS

A total of 120 isolates identified as *E. coli* by conventional methods were confirmed by MALDI‐TOF MS method spectra were identified using the updated IVD database containing 10,694 main spectrum profile (MSPs). For microbial biomass analysis, one colony was placed on a special steel 96 microdiscovery plate (MSP) and spread as a thin film in the wells. Then 1 µL of matrix solution (12.5 mg/mL matrix in a mixture of 50% ACN and 2.5% TFA) was added and allowed to dry completely at room temperature after drying. The system was operated in the MALDI‐TOF MS instrument in linear positive ion mode with the optimised method for the identification of microorganisms. A 60 Hz nitrogen laser at 337 nm was used as an ion source. Each sample was examined three times by applying laser pulses consisting of 40 packets of 240 pulses in the measurement of each colony, and the highest readings were included in the analysis. Besides 120 isolates, the standard strain *E. coli* ATTC 25922 was also examined. Bruker BTS was used for internal quality control of the mass spectrometer

### 2.3 The Use of Principal Component Analyses in MALDI‐TOF MS

Spectra were analysed using MALDI Biotyper Flex Analysis version 3.4, and MALDI Biotyper 3.1 database (Bruker Daltonics). Biotyping analysis using MALDI‐TOF MS reveals the characteristic mass and peak intensity distributions of ribosomal 16S proteins in a sample. This mass spectrum represents a ‘molecular fingerprint’ as it is species specific for many microorganisms (Kostrzewa and Maier 2017). All identification score criteria used were applied in accordance with the manufacturer's recommendations (Bruker). The analysis of the spectra was performed using the PCA method using MATLAB software. This method created clustered spectra groups with similar variational features and helped visualise the differences between them based on the unique peptide and protein peaks in each spectrum. This method also reduced the dimensionality of the data set while representing the data in a three‐dimensional coordinate system.

The variance among all bacteria was automatically calculated with the software support. In order to investigate the variance differences detected among *E. coli* strains, matches were made with the reference MSP of *E. coli* ATCC 25922 THL strain in the database.

In addition, virtual gel images (VGIs) containing the projection of peaks in the spectra of *E. coli* isolates were created. In these gel images, vertical VGI traces with varying relative abundance values on the green background correspond to each peak in the spectrum. These vertical VGI traces are represented by a variation from low relative abundance (light white) to high relative abundance (thick white). For cluster analysis, PCA dendrograms and two‐dimensional (2D) scatter plots were created. In addition, the similarity (closeness) and difference (distance) relationships of each bacterium were determined statistically by the composite correlation index (CCI).

### 2.4 In Vitro Antibiotic Susceptibilities in *E. coli* Isolates

Antibiotic susceptibilities of the isolated *E. coli* were investigated via disc diffusion method. For determination of antibiotic susceptibility, ampicillin (AMP; 10 µg), cefazolin (CZ; 30 µg), ceftazidime (CAZ; 10 µg), cefotaxime (CTX; 5 µg), ceftriaxone (CTR; 30 µg), ciprofloxacin (CIP; 5 µg), gentamycin (GEN; 10 µg), amikacin (AK; 30 µg), trimethoprim/sulfamethoxazole (SXT; 25 µg), imipenem (IPM; 10 µg) and meropenem (MEM; 10 µg) discs were used. The *E. coli* ATCC 25922 strain was used as a control.

### 2.5 Determination of ESBL in *E. coli* Isolates

#### 2.5.1 Double‐Disc Synergy Test

With respect to the antibiotic susceptibility results obtained via the disc diffusion method, a double‐disc synergy test was performed to investigate the ESBL positivity in bacterial isolates that were moderately susceptible or resistant to one or both CTX and CAZ according to EUCAST limit values.

A bacterial suspension with a turbidity of 0.5 McFarland was prepared from an overnight fresh bacterial culture and spread on the surface of MHA medium. An amoxicillin‐clavulanic acid disc (AMC 20/10 µg) was placed in the centre of the Petri dish, and CTX (30 µg), CAZ (30 µg) and cefepime (FEB; 30 µg) discs were placed around it at a distance of 15–30 mm from the centre. The Petri dishes were then incubated at 37°C for 18–24 h. The appearance of plaques in which the zone diameter of any of the cephalosporin (CTX, CAZ, or cefepime) discs was enlarged on the side facing the amoxicillin‒clavulanic acid disc, or a keyhole‐like image in which no growth was observed, was considered positive (EUCAST 2022).

#### 2.5.2 Combined Disc Method

The combined disc method was applied to isolates with uncertain ESBL presence as a result of the double‐disc synergy method. Bacterial suspensions adjusted to 0.5 McFarland turbidity were prepared from fresh cultures and inoculated on MHA media. Cefepime (30 µg) discs with and without clavulanic acid (10 µg) were placed on the plates. The zone diameters around the discs with and without clavulanic acid were compared after overnight incubation at 37°C. A zone diameter around the combination disc ≥5 mm larger than the zone diameter of the disc without clavulanic acid was considered ESBL positive.

### 2.6 Whole‐Genome Sequence Analysis of ESBL‐Positive *E. coli*

Whole‐genome analysis was performed on a single ESBL‐positive isolate. DNA extraction was performed via the EZ1&2 Virus Mini Kit (Qiagen, Germany). The extracted DNA was quantified on a Qubit 4.0 fluorometer (Invitrogen, Singapore) via a dsDNA high‐sensitivity assay kit (Life Technologies, USA) following the manufacturer's instructions. To prepare the library, a barcoded sequencing library was prepared, and whole‐genome sequencing was performed on 700 ng of extracted DNA via the Native Barcoding Genomic DNA Kit (SQK‐LSK109)‐Oxford Nanopore Technologies (ONT, UK). The library was loaded onto FLO‐MIN106D flow cells on a GridION instrument (ONT, UK). Sequence data were analysed via bioinformatics tools, including Flye, Blast + Minimap2 and Geneious Prime.

### 2.7 Bioinformatic Analysis

After sequencing, the data is obtained in FAST5 format and converted into FASTQ format using Dorado (v0.5.1, Oxford Nanopore Technologies) with the high‐accuracy model. Reads in FASTQ format are assembled into longer contigs using Flye (de novo assembly method). The long contigs generated through de novo assembly are analysed using Local BLAST (version 2.14.0+) to identify the best species match. Various parameters, including query coverage, pairwise identity, bitscore and e‐value, are examined in the BLAST results for each sample to identify the organism with the highest similarity. Genomes of closely related species and strains of the identified organism are retrieved from GenBank in .gb format.

The raw reads in FASTQ format and the reference genomes are imported into Geneious Prime (v2023.2.1). The reads are aligned to the reference genomes, and the organism with the best alignment statistics for a sample is selected for further analysis. To detect polymorphic structures such as single nucleotide polymorphisms (SNPs), insertions/deletions (INDELs) and amino aid changes, and the ‘Find variations/SNPs’ tool in Geneious Prime is used. A consensus genome is generated using the ‘Generate Consensus Genome’ tool.

To identify the functional genes in the consensus genome, such as CDS, tRNA, rRNA and ncRNA regions, the ‘Annotate from Database’ tool in Geneious Prime is used, with the GenBank reference genomes serving as the database for annotation. Antimicrobial resistance genes in the consensus genome are detected using the CARD database (https://card.mcmaster.ca/). Similarly, virulence and pathogenicity genes are identified using the VFDB database (http://www.mgc.ac.cn/VFs/).

## 3 Results

### 3.1 Identification of Isolates

*E. coli* was isolated from a total of 120 samples, 78 from soil and 42 from faeces. Table 1 shows the number of samples collected.

**TABLE 1:** Number of collected samples and *E. coli* strains isolated by district.

Farm Location	Soil samples	Faecal samples	Total samples	*E. coli* isolated from soil samples	*E. coli* isolated from faecal samples	Total *E. coli* isolates
1^*^	61	18	79	25	17	42^**^
2^*^	41	15	56	24	15	39^**^
3^*^	39	10	49	29	10	39^**^
Total	141	43	184	78	42	120

* 1, Golbasi; 2, Cubuk; 3, Haymana.

** The 42 *E. coli* isolates from 18 soil and 7 faeces samples of 8 cattle farms, 3 soil and 5 faeces samples of 4 sheep farms, 4 soil and 5 faeces samples of 5 poultry farms of Golbasi. The 39 *E. coli* isolates from 2 soil and 6 faeces samples of 3 cattle farms, 8 soil and 3 faeces samples of 2 sheep farms, 15 soil and 5 faeces samples of 5 poultry farms of Cubuk. The 39 *E. coli* isolates from 8 soil and 3 faeces samples of 3 cattle farms, 10 soil and 4 faeces samples of 3 sheep farms, 11 soil and 3 faeces samples of 5 poultry farms of Haymana district.

### 3.2 Identification of Bacteria by MALDI‐TOF MS

**FIGURE 1:**
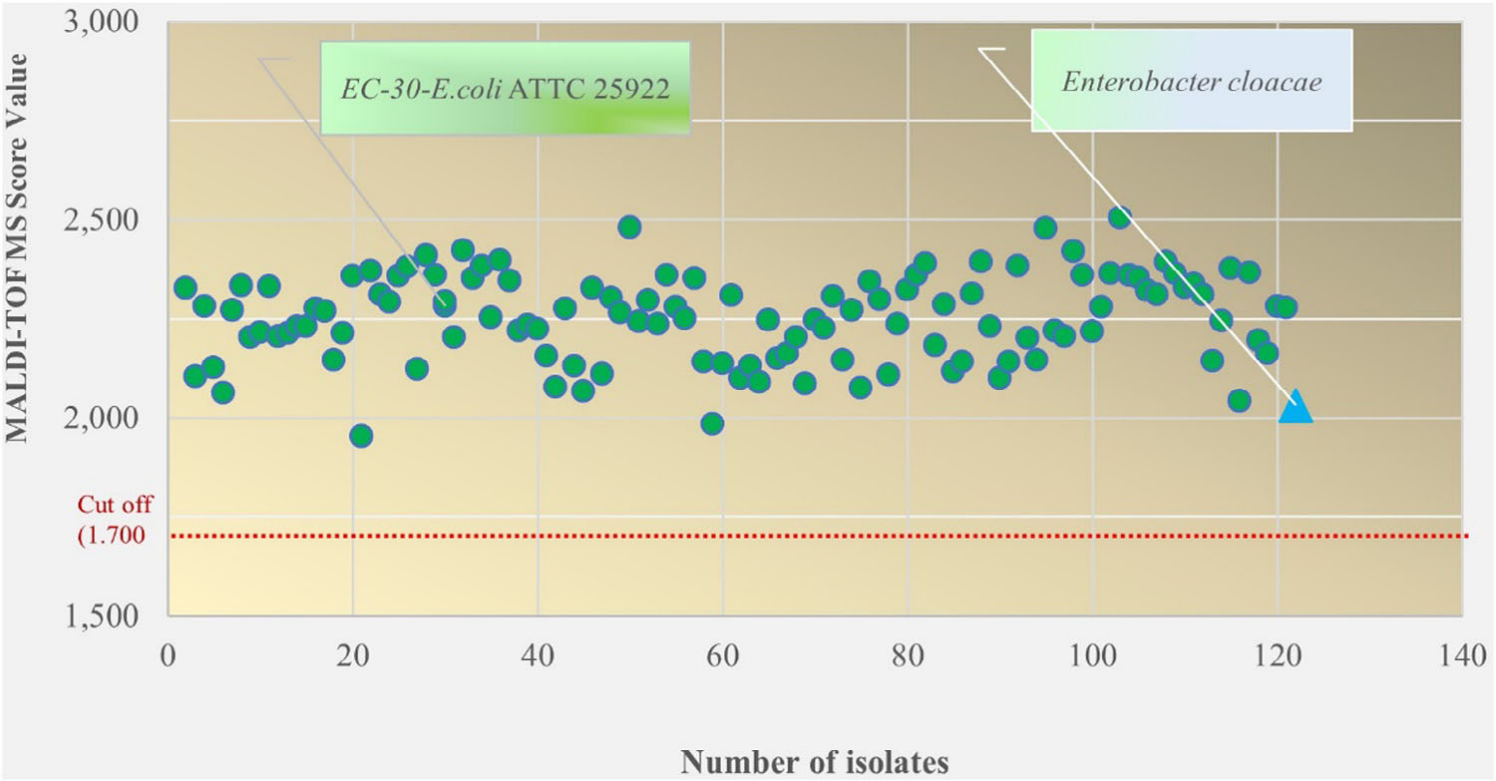
Distribution of the MALDI‐TOF MS scores of the isolates defined as *E. coli* (*n* = 120) and *E. cloacae* (*n* = 1).

Among the 120 isolates isolated from soil and faeces, 120 were identified as *E. coli* (EC), and only one was identified as *Enterobacter cloacae*. The distribution of the identification scores of all the isolates is presented in Figure 1. As shown in Figure 1, the identification score values were mostly between 2.000 and 2.503 (except for two isolates). There was no definition under the cut‐off (1.700) value for species identification.

**FIGURE 2:**
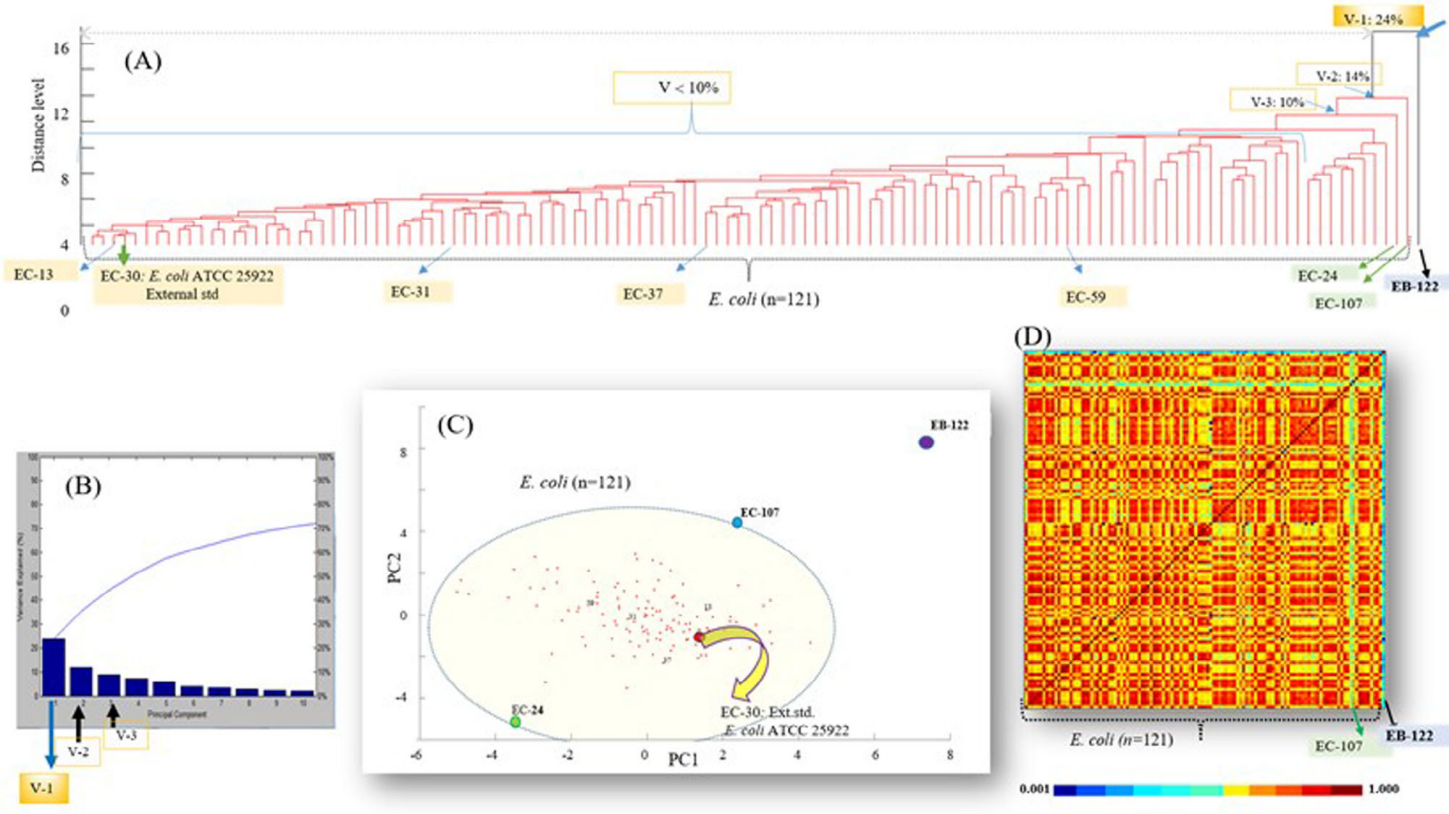
MALDI‐TOF MS‐based PCA of 120 isolates isolated from soil and faeces. (A) Dendrogram profile. (B) Variance explained. (C) 2D scatter plo t. (D) CCI‐colour matrix of all the isolates. EB, *Enterobacter cloacae*; EC, *Escherichia coli*; V, variance.

When MALDI‐TOF MS‐based PCA analyses of all the isolates were performed, it was observed that *E. cloacae* occurred in a separate line in the dendrogram profile, whereas the external standard strains (*E. coli* ATTC 25922) and *E. coli* isolates (*n* = 120) were located in a large cluster (Figure 2A). When the variance profile in Figure 2B was examined, the first three of the 10 variance (PC) values were 24% (PC1), 14% (PC2) and 10% (PC3), whereas the other variances ranged from <10% to ≥2%. As expected, the first variance belonged to PC1 (24%) *E. cloacae*, whereas the others (from PC2 to PC10) belonged to all *E. coli* cluster members (*n* = 120). Figure 2A, C shows that PC2 (14%) belongs to EC‐107 and that PC3 (10%) belongs to EC‐24. When the dendrogram profile in Figure 2A was examined in detail, EC‐107 was located on a separate line from the large cluster with a 14% variance value, whereas the EC‐24 isolate was located with a 10% variance. In the 2D scattering profile, where each dot represents a spectrum of an isolate, as presented in Figure 2C, the EB isolate scattered the farthest as expected when viewed relative to the zero‐origin point. In addition, EC‐24 and EC‐107 were scattered farther relative to cluster members. In addition to a total of 120 isolates, the analysed *E. coli* ATTC 25922 standard strain was located in the leftmost region of the dendrogram profile (Figure 2A), with the lowest variance value (2%) compared with the cluster members. Consistent with this, it also showed scattering within the cluster and close to the zero line in the 2D‐scattering profile. As shown in Figure 2D, after CCI analysis of all the isolates (*n* = 122) was performed, the CCI colour matrix was created. In this colour matrix, each box corresponds to a strain. The proximity index value (maximum: 1.000; 100%) ranges from dark red to dark blue (minimum: 0,001; 01%). Accordingly, in the projection of each isolate to itself, the index colour is dark red and the index value is 1 (100%). The index values corresponding to the colours in the CCI matrix ranged from 0% to 39% for dark blue to turquoise, from 40% to 59% for light green to dark green and finally, from yellow to dark red, which corresponded to CCI values ranging from 60% to 100%.

**FIGURE 3:**
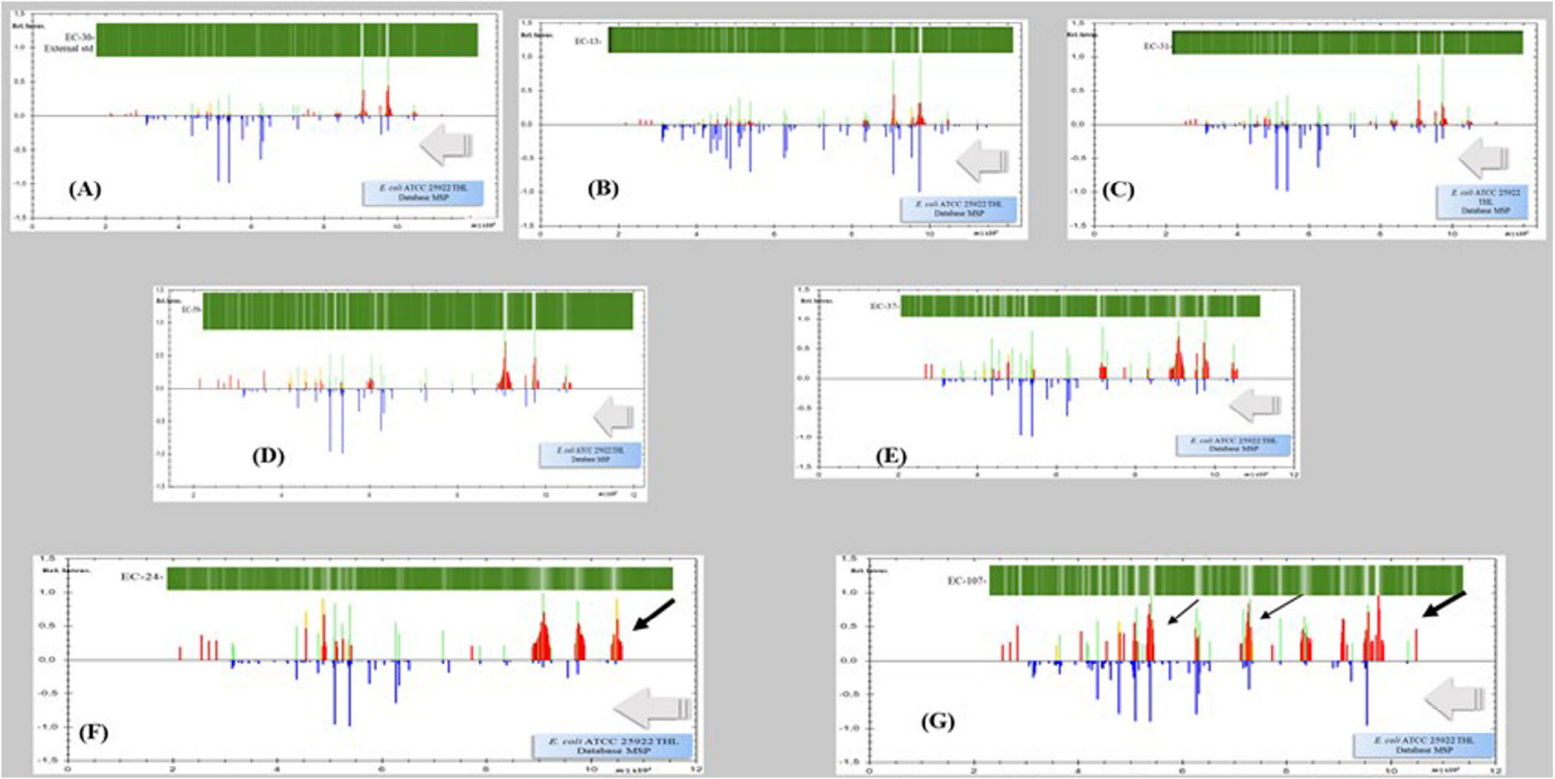
Matching the spectra of some EC isolates with database‐MSP (*E. coli* ATTC 25922 THL) to investigate the variance values. (A) External standard strain (*E. coli* 25922; 2% variance), (B) EC‐13 isolate (2% variance), (C) EC‐31 isolate (2% variance), (D) EC‐59 isolate (2% variance), (E) EC‐37 isolate (2% variance), (F) EC‐24 isolate (10% variance) and (G) EC‐107 isolate (14% variance).

Figure 3 shows the results of the spectral matching of some EC strains and EC‐30, which was used as an external standard for *E. coli* ATTC 25922, with the reference MSP of *E. coli* ATTC 25922 THL in the MALDI Biotyper 3.1 database. The mass/charge ratio is shown on the x‐axis, and the relative abundance (Rel. intens.; RI) of the values of the peptides and proteins are shown on the y‐axis. While the (+) RI values in each spectral pattern belong to strains, the (−) RI values belong to the *E. coli* ATTC 25922 THL MSP found in the database. In addition to the MSP maps of each strain presented in Figure 3, VGI images of these strains were also included.

An important detail when evaluating matches with the database‐MSP (+) RI value was the interpretation of the representative vertical colours in the spectra of the isolates in the second part. Accordingly, the green bar indicates a one‐to‐one match with the MSP, the yellow‒orange bar indicates an acceptable level of match, and the red bar indicates no match. The evaluation revealed that the *E. coli* isolates presented in Figure 3 generally matched the database MSP (*E. coli* ATTC 25922 THL).

However, when the spectral matching profiles in Figure 3 are examined in detail, although there are matches with the main peaks, there are also some differences. At this stage, it is necessary to understand which peaks of each isolate are dominant and which different peaks they contain according to the database MSP (*E. coli* ATTC 25922 THL). For this purpose, VGIs of each strain were created. In these VGIs with vertical white lines on a green background, high (≥20%) peptide and protein peaks (HIPP) of high intensity are represented by thick white vertical lines, and these lines seem to fade as the intensity decreases. The inclusion of these VGI images revealed traces of peptides and proteins from each strain presented in Figure 3.

Figure 3A shows the database‐MSP mapping of the external standard strain coded EC‐30. As expected, the mapping of the EC‐30 standard strain (*E. coli* ATTC 25922) to the MSP database *E. coli* ATTC 25922 THL appears to be fairly consistent, with mostly green bars in general.

The four isolates (EC‐13, EC‐31, EC‐37 and EC‐59) presented in Figure 3 (B, C, D and E, respectively) had low variance values (2%–10%) and were randomly selected from the large EC cluster. As shown in the dendrogram and scattering profile (Figure 2A,C), these isolates were located in small clusters with subfractions in the large cluster. In addition, HIPPs in the VGI images of these four isolates were mostly between 9000 and 9800 Da, similar to those of the EC‐30 standard strain (*E. coli* ATTC 25922). In addition, database‐MSP matches of these isolates, such as the EC‐30 standard strain (*E. coli* ATTC 25922), also appear to be generally compatible.

As mentioned above, although they are members of the large EC cluster, both EC‐24 and EC‐107 were partially separated from the cluster with 10% and 14% variance, respectively, in the dendrogram profile in Figure 2A. The matches of these strains with database‐MSP data are presented in Figure 3F and Figure 3G, respectively. The red bars are mostly between 9000 and 12000 Da in the spectral match of EC‐24 in Figure 3F, whereas the red bars are more common than those of EC‐24 between 5000 and 12000 Da in the profile of EC‐107.

Although the peptides and proteins in the spectra of these two strains match those in the database MSP, more peptides and proteins outside the current database pattern were identified. This situation causes the variance values to be slightly different than those of the other cluster members.

### 3.3 Antibiotic Susceptibility of *E. coli* Isolates

Antibiotic susceptibility testing revealed that 120 *E. coli* isolates were resistant to AMP 12.5% (15), trimethoprim/SXT 6.6% (8), cefazolin 0.83% (1), CIP 2.5% (3), CAZ 0.83% (1), CTX 1.6% (2) and CTR 1.6% (2). One hundred percent (120) of the bacteria were susceptible to the antibiotics gentamicin, amikacin, IPM and MEM. The results of the antimicrobial sensitivity tests are given in Table 2.

**TABLE 2:** Results of the antimicrobial susceptibility test.

Antibiotics	Resistant (%)	Intermediate (%)	Sensitive (%)
Ampicillin	15 (12.5)	–	105 (87.5)
Trimethoprim/sulfamethoxazole	8 (6.6)	1 (0.83)	111 (925)
Cefazolin	1 (0.83)	119 (99.1)	–
Ciprofloxacin	3(2.5)	–	117 (97.5)
Gentamicin	–	–	120 (100)
Ceftazidime	1(0.83)	–	119 (99.1)
Cefotaxime	2 (1.6)	–	118 (98.3)
Ceftriaxone	2 (1.6)	–	118 (98.3)
Amikacin	–	–	120 (100)
Imipenem	–	–	120 (100)
Meropenem	–	–	120 (100)

### 3.4 ESBL Results

According to the antibiotic susceptibilities determined by the disc diffusion test, only isolate H2 (isolated from the soil samples of the poultry farms in third district) was found to be positive as a result of the double‐disc synergy test. The combined disc method was applied to isolates where the presence of ESBL was uncertain as a result of the double‐disc synergy test. No ESBL‐positive isolates were found via the combined disc method.

### 3.5 Whole‐Genome Sequencing Analysis of ESBL‐Positive *E. coli*

**FIGURE 4:**
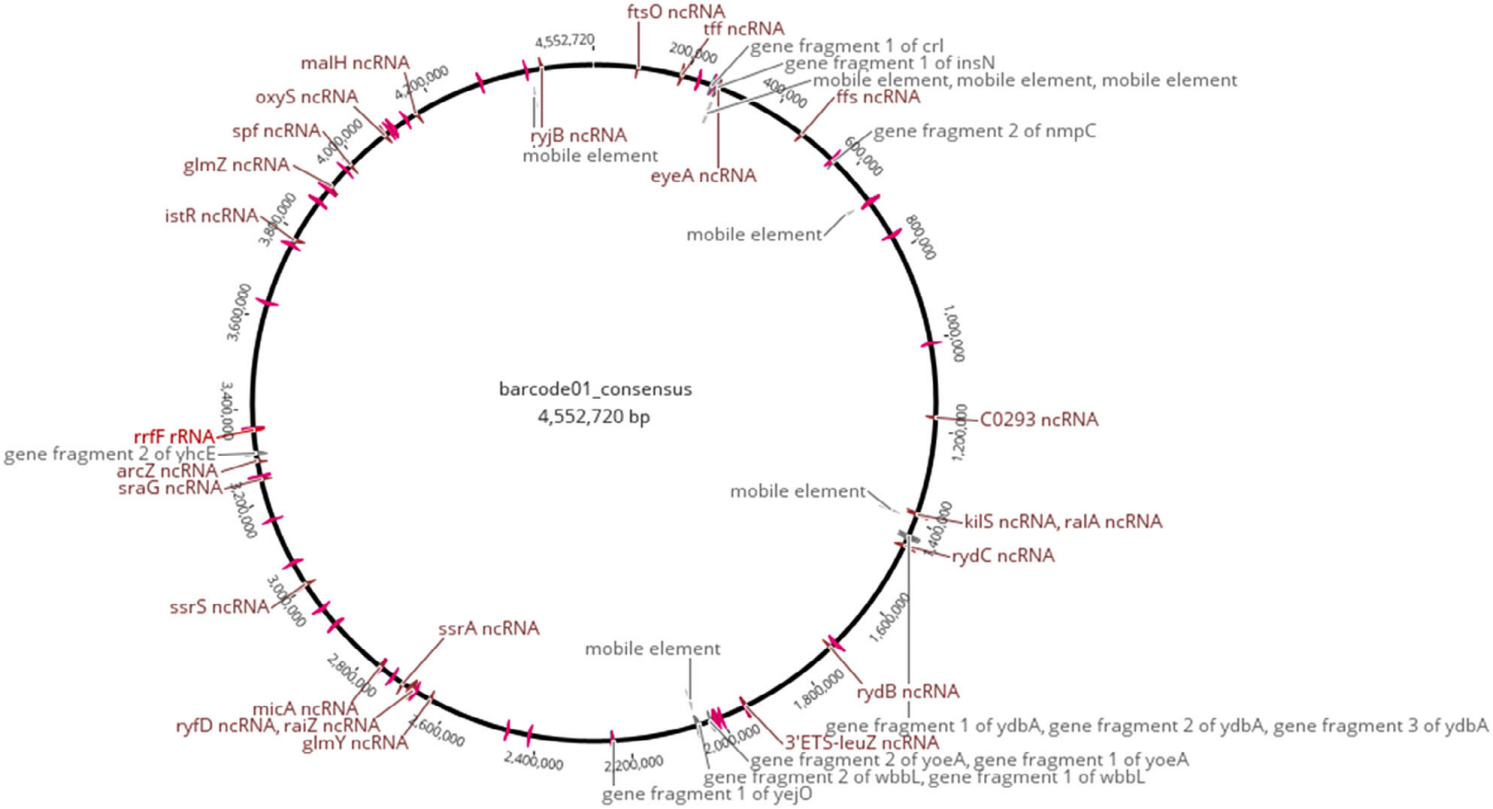
Circular representation of the consensus genome resulting from the alignment of the bacterial isolate H2 to the reference genome of *Escherichia coli* str. K‐12.

The DNA concentration was determined to be 0.440 ng/µL. A total of 166,700 reads were obtained from the bacterial whole‐genome sample sequenced with Oxford Nanopore Technologies. The reads were aligned to the *E. coli* reference genomes available at NCBI with Minimap2, an alignment tool for long reads, via the Geneious Prime (v2022.2.2) application. As a result of the alignment, the reads obtained from the sample were best aligned to the *E. coli* str. K‐12 (NCBI GenBank ID: NC_000913.3) strain (Figure S1). The coverage rate of the reads to the reference genome was 93.1%, the pairwise similarity rate to the reference genome was 88.8%, and the average coverage value was 153X. In addition, the pairwise similarity of the bases to the reference genome was 78.3%. Figure 4 shows the annotated circular genome of the linear consensus genome generated by aligning the ‘Barcode01 (H2)’ sample to the reference genome of *E. coli* str. K‐12. The genome sequences of the H2 strains have been deposited at GenBank under the accession number PRJNA1191815.

We screened virulence‐associated genes in the H2 strain (Table S1). H2 strains carry Type I fimbriae genes (*fimB, fimC, fimD*, *fimE, fimF, fimG, fimH and fimI*) responsible for colonisation; haemorrhagic *E. coli* pilus genes (*hcpA, hcpB and hcpC*) responsible for conjugation; *E. coli* common pilus genes (*ecpA, ecpC, ecpD, ecpE and ecpR*); and curli fibre genes (*cgsD, cgsF, cgsG, cgsA, cgsB and cgsC*) responsible for adhesion to surfaces, cell aggregation and biofilm formation.

The consensus whole‐genome sequence generated via alignment to *the E. coli* K‐12 subspecies was analysed via the RGI algorithm using the ARDB database, and genes related to antimicrobial resistance were identified. The AMR gene family, drug class, resistance mechanism and similarity percentage of the matched region are shown in Table S2. The most common resistance mechanism involves an antibiotic efflux pump. The *acrA, mdtH, marA, evgA* and *evgS* genes were detected for fluoroquinolone resistance by efflux pump. We also identified the *emrE, H‐NS, evgA* and *evgS* genes, which are associated with macrolide antibiotic resistance, and the *mdfA, emrY, emrK, evgA* and *evgS genes*, which are associated with tetracycline antibiotic resistance.

The plasmids carried by the H2 bacterial isolate and the antimicrobial resistance genes of these plasmids were searched in the plasmid database. Table S3 shows the plasmids found and the antimicrobial resistance genes of the plasmids. The plasmids carried by strain H2 were found to be similar to those of NZ_KR827684 and NZ_CP010149. Accordingly, strain H2 carries the *CTX‐M‐14* and *TEM‐1* genes, which are responsible for resistance to beta‐lactam antibiotics and may be responsible for the transmission of this resistance through its plasmids. The H2 strain also carried the *sul1, sul2* and *sul3* genes, which are responsible for sulfonamide resistance through its plasmids. The H2 strain was found to carry the resistance genes *tet(A), tet(B)* (tetracycline resistance); *aadA2, APH(3'')‐Ib, APH(6)‐Id, aadA5* (aminoglycoside resistance); and *mphA* (macrolide resistance) via plasmids.

## 4 Discussion

Antimicrobial resistance is a major threat to both human and animal health. In addition to their use in human medicine, the majority of antibiotics produced worldwide are used in veterinary medicine and animal husbandry. In livestock and aquaculture, antibiotics are used to prevent and treat infections and promote growth. As their use as growth factors is considered to be an important factor in the spread of antibiotic resistance, their use for this purpose has been banned in our country since 2006. The classes of antibiotics used to treat humans and animals are similar, so the emergence and spread of antibiotic‐resistant bacteria are increasing (Filazi, Dikmen, and Kuzukıran 2015; Roca et al. 2015; Süleymanoğlu, Aksu, and Aydın 2022; Töreci 2003).

Antibiotic‐resistant bacteria can be transmitted to humans through direct contact with antibiotic‐resistant microorganisms and the food chain or indirectly through environmental contamination of farm waste. Animals can be contaminated with faeces during slaughter, or contaminated water can be used to grow crops. Another risk is the transmission through cross‐contamination of food (Roca et al. 2015; Verraes et al. 2013).

ESBL is an important issue in antibiotic resistance. ESBL‐positive bacteria are also multidrug resistant. The CDC states that ESBL‐positive Gram‐negative bacilli are a major health threat. In addition, ESBL should be analysed with a One Health approach (Süleymanoğlu, Aksu, and Aydın 2022). There are studies on the isolation of *E. coli* from various sources in the literature. Debbarma et al. (2020) collected a total of 420 samples, including 245 faeces (52 of which were diarrhoeal) and 175 meat samples from 3 food animal categories, namely, cattle, pigs and poultry and *E. coli* was isolated from 66 (15.71%) samples. Mukuna et al. (2023) isolated 113 (76.4%) *E. coli* strains from a total of 285 environmental samples (manure, soil and water) from cattle and goat farms. Marijani (2022) analysed 92 fresh fish samples from open‐air fish markets and supermarkets in Tanzania. He identified seven different species of bacteria, and the most common species was *E. coli*, with 36 (39%).

In our study, a total of 184 soil and faeces samples, from cattle, sheep and poultry, were collected from farms and 120 (65.2%) *E. coli* were isolated. These isolates were then confirmed via MALDI‐TOF MS. MALDI‐TOF MS‐based PCA analyses of all identified strains were performed under the guidance of the *E. coli* ATTC‐25922 standard strain. Accordingly, extremely significant results were obtained in the variance calculations among the 120 isolates, including *E. cloacae* from the *Enterobacteriaceae* family. Accordingly, it is clearly separated from *E. coli* strains with 24% variance since it is from the same family. Rather, our main aim in this study was to investigate whether there are significant differences between *E. coli* strains in terms of peptide and protein profiles. For this purpose, the closeness and distance indices of each *E. coli* strain to each other were evaluated. *E. coli* strains are generally very similar to each other, except for EC‐107, which differs, albeit partially, from each other. Matching the spectra of the *E. coli* ATTC 25922 THL reference strain and the spectra of several *E. coli* (EC‐13, EC‐24, EC‐31, EC‐37, EC‐59 and EC‐107) strains in the Biyotper 3.1 (Bruker) database, when evaluated under the guidance of the *E. coli* ATTC 25922 external standard strain, it was determined that they were compatible with each other in terms of basic peaks.

The reason why *E. coli* cluster members have small variance values is that *E. coli* bacteria, which have a wide variety in nature, show partial differences in their peptide‒protein content to adapt to nature, as expected. Distinguishing these small differences very sensitively via MALDI‐TOF MS‐based PCA analyses will make significant contributions to the evaluation of microbiological analysis results.

*E. coli* isolates from the soil and faecal samples were tested for susceptibility to 11 different antibiotics. No resistance to the antibiotics gentamicin, amikacin, IPM and MEM was detected. The highest intermediate susceptibility rate was 99.1% for cefazolin (119). In addition, resistance was detected in a total of 17 *E. coli strains*. Of these, 12 were *E. coli* isolates from soil, and 5 were *E. coli* isolates from faecal samples.

Sonola et al. (2021) collected a total of 960 samples from chickens (236), humans (243), rodents (101) and soil (290) in Tanzania and detected *E. coli* in 650 (67.7%) of these samples. The antibiotic resistance rates of the isolates were as follows: IPM (79.8%), CTX (79.7%), tetracycline (73.7%), amoxicillin/clavulanate (49.4%), CIP (40.2%) and gentamicin (9.7%). In addition, multidrug resistance was observed in 78.8% of all the isolates.

In the study conducted in Thailand, a total of 587 samples were collected, including 467 faeces samples from poultry (426 chickens and 41 ducks), 88 samples from the farm environment (54 soil and 34 water) and 32 faeces samples from farmers. 159 isolates were found to be ESBL producers and were isolated from 50% of human faeces samples, 25.9% of poultry (24.9% chicken and 36.6% duck) and 25% of environmental samples (soil 16.7% and water 38.2%). Among the 159 ESBL‐producing *E. coli* isolates, 152 were positive for *bla*_CTX‐M_ (Tansawai, Walsh, and Niumsup 2019).

In Egypt, 46.6% (98) and 28.6% (16) of ESBL‐producing *E. coli* were found in 210 rectal swabs and 56 culture and environmental samples from dairy farms, respectively. A total of 114 (42.8%) isolates were identified as ESBL‐producing *E. coli*, each of which carried at least one of the ESBL genes (*bla*_CTX‐M15_, *bla*_CTX‐M9_, *bla*_TEM_ or *bla*_SHV_). A total of 103 (90.4%) isolates contained the *bla*_CTX‐M1/15_ gene, 89 (78%) isolates contained the *bla*_TEM_ gene, 6 (5.2%) isolates contained the *bla*_CTX‐M9_ gene and 1 (0.87%) isolate contained the *bla*_SHV_ gene (Braun et al. 2016).

Although broiler facilities were not included in our study, the literature has shown that these facilities may also be a source of resistance genes. Some studies have reported that it is a critical control point in poultry production that may affect public health as well as farm animals. In a study conducted in Greece, *mcr‐1* gene carriage was detected only in broiler isolates (Xexaki et al. 2023).

Whole‐genome analysis of the H2 bacterial isolate revealed the presence of the ESBL genes CTX‐M‐14 and TEM‐1 on the plasmids. In addition, the aadA2, APH(3'')‐Ib, APH(6)‐Id, AAC(3)‐IId, aadA5, APH(3'')‐Ib and APH(6)‐Id genes, which are responsible for resistance to aminoglycoside antibiotics; the *tet*(A) and *tet*(B) genes, which are responsible for resistance to tetracycline antibiotics; and the *sul*1, *sul*2 and *sul*3 genes, which are responsible for resistance to sulfonamide antibiotics, were found. The chromosomal genes *acr*A and *mar*A, which promote the excretion of antibiotics such as cephalosporins and tetracyclines and reduce membrane permeability to antibiotics, were found to be responsible for the multidrug resistance phenotype.

## 5 Conclusion

Compared with those of clinical isolates, the antibiotic resistance rates of the environmental isolates analysed in our study were low. We believe that this is because the use of antibiotics as growth promoters in animals was banned in 2006. However, when the plasmids carried by the ESBL‐positive isolate isolated in our study and the resistance genes in these plasmids are examined, there is still a need for comprehensive studies to determine antibiotic sensitivities and resistance genes in environmental samples with One Health perspective.

## Author Contributions

**Didem Karademir**: Investigation; methodology; visualisation; writing—original draft preparation. **Banu Kaskatepe**: Conceptualisation; data curation; supervision; writing—review and editing. **Hilal Basak Erol**: Investigation; methodology; visualisation; writing—original draft preparation. **Suleyman Yalcin**: Methodology; validation; visualisation; writing—original draft preparation. **Yasemin Numanoglu Cevik**: Methodology; validation; visualisation; writing—original draft preparation. All authors have read and agreed to the published version of the manuscript.

## Conflicts of Interest

The authors declare no conflicts of interest.

### Peer Review

The peer review history for this article is available at https://publons.com/publon/10.1002/vms3.70209.

## Supporting information



Supporting Information

## Data Availability

The data that support the findings of study are available from the corresponding author upon responsible request.
