# Supplementary material for: The Study of Escherichia coli as Antimicrobial‐Resistant Sentinel Microorganism Isolated in the Farms of Three Districts of Ankara by MALDI‐TOF MS and Genomic Analysis
Source: Vet Med Sci. 2025 Apr 2;11(3):e70209. doi: 10.1002/vms3.70209 (PMC11964152; doi:10.1002/vms3.70209)
Supplement: Supplementary file 1 — Supporting Information [file VMS3-11-e70209-s001.docx]

**The study of *E. coli* as antimicrobial resistant sentinel microorganisms in the farms by Maldi-TOF MS and genomic analysis**


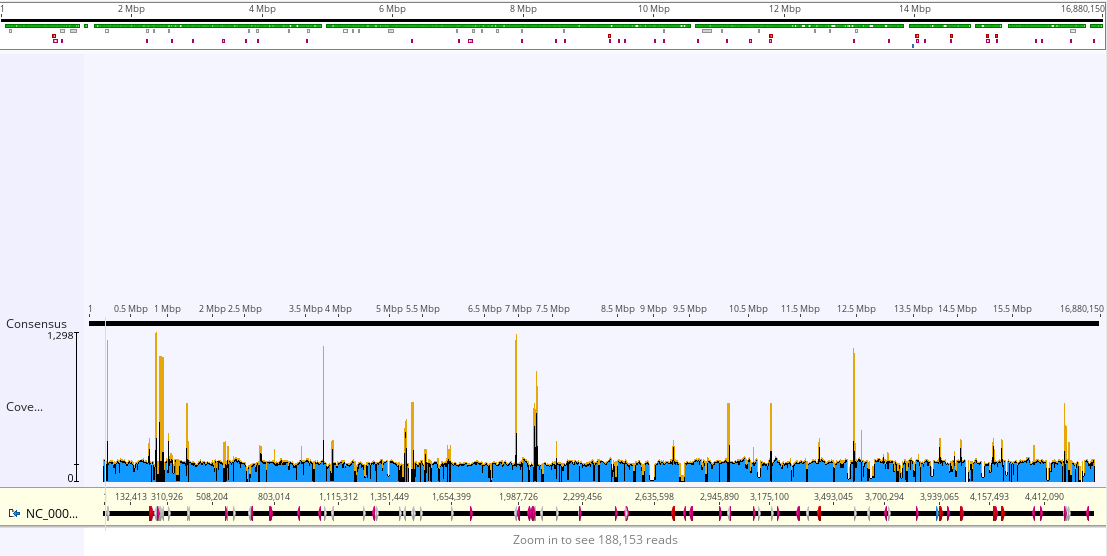


**Figure S1.** Coverage image of the genome aligned to the reference genome of *Escherichia coli* str. K-12 (NCBI GenBank ID: NC_000913.3). The blue colored areas in the alignment image show the density and coverage of the reads in the genome, the aligned reads cannot be shown in the image due to the large number of aligned reads.

**Table S1.** Virulence factors of H2 strain

| Virulence Factors | Virulence-Associated Genes | ORF | Function |
| --- | --- | --- | --- |
| Curli fibers | *cgsD* | orf01073 | - |
| Curli fibers | *cgsF* | orf01070 | - |
| Curli fibers | *cgsG* | orf01069 | - |
| Curli fibers | *csgA* | orf01076 | - |
| Curli fibers | *csgB* | orf01075 | - |
| Curli fibers | *csgC* | orf01077 | - |
| *E. coli* common pilus (ECP) | *ecpA* | orf00299 | ECP, composed of a 21-kDa pilin subunit EspA, is a pilus-adherence factor that is crucial to the virulence of E. coli O157 in humans, and is also carried by commensal strains of E. coli. It is suggested that pathogenic E. coli strains may use ECP to mimic commensal E. coli and provide themselves with an ecological advantage for host colonization and evasion of the immune system. |
| *E. coli* common pilus (ECP) | *ecpC* | orf00296  orf00297 | ECP, composed of a 21-kDa pilin subunit EspA, is a pilus-adherence factor that is crucial to the virulence of E. coli O157 in humans, and is also carried by commensal strains of E. coli. It is suggested that pathogenic E. coli strains may use ECP to mimic commensal E. coli and provide themselves with an ecological advantage for host colonization and evasion of the immune system. |
| *E. coli* common pilus (ECP) | *ecpD* | orf00295 | ECP, composed of a 21-kDa pilin subunit EspA, is a pilus-adherence factor that is crucial to the virulence of E. coli O157 in humans, and is also carried by commensal strains of E. coli. It is suggested that pathogenic E. coli strains may use ECP to mimic commensal E. coli and provide themselves with an ecological advantage for host colonization and evasion of the immune system. |
| *E. coli* common pilus (ECP) | *ecpE* | orf00294 | ECP, composed of a 21-kDa pilin subunit EspA, is a pilus-adherence factor that is crucial to the virulence of E. coli O157 in humans, and is also carried by commensal strains of E. coli. It is suggested that pathogenic E. coli strains may use ECP to mimic commensal E. coli and provide themselves with an ecological advantage for host colonization and evasion of the immune system. |
| *E. coli* common pilus (ECP) | *ecpR* | orf00300 | ECP, composed of a 21-kDa pilin subunit EspA, is a pilus-adherence factor that is crucial to the virulence of E. coli O157 in humans, and is also carried by commensal strains of E. coli. It is suggested that pathogenic E. coli strains may use ECP to mimic commensal E. coli and provide themselves with an ecological advantage for host colonization and evasion of the immune system. |
| *E. coli* laminin-binding fimbriae (ELF) | *elfA* | orf00970 | - |
| *E.coli* laminin-binding fimbriae (ELF) | *elfG* | orf00975 | - |
| Hemorrhagic *E. coli* pilus (HCP) | *hcpA* | orf00106 | - |
| Hemorrhagic *E. coli* pilus (HCP) | *hcpB* | orf00105 | - |
| Hemorrhagic *E. coli* pilus (HCP) | *hcpC* | orf00104 | - |
| Type I fimbriae | *fimB* | orf04560 | Facilitates colonization |
| Type I fimbriae | *fimC* | orf04565 | Facilitates colonization |
| Type I fimbriae | *fimD* | orf00142  orf00547  orf01581  orf02231  orf03327  orf04566 | Facilitates colonization |
| Type I fimbriae | *fimE* | orf04561 | Facilitates colonization |
| Type I fimbriae | *fimF* | orf01580  orf04567 | Facilitates colonization |
| Type I fimbriae | *fimG* | orf01579  orf04568 | Facilitates colonization |
| Type I fimbriae | *fimH* | orf04569 | Facilitates colonization |
| Type I fimbriae | *fimI* | orf04563 | Facilitates colonization |
| Invasion of brain endothelial cells (Ibes) | *ibeB* | orf00582 | Contributes to brain microvascular endothelial cells (BMECs) invasion |
| Invasion of brain endothelial cells (Ibes) | *ibeC* | orf04184 | Contributes to brain microvascular endothelial cells (BMECs) invasion |
| Salmochelin siderophore | *iroN* | orf00597 | Iron uptake |
| EspL1 | *espL1* | orf01820 | - |
| EspL4 | *espL4* | orf04238 | - |
| EspR1 | *espR1* | orf01542 | - |
| EspX4 | *espX4* | orf04261 | - |
| LEE locus encoded TTSS | *escV* | orf02002 | - |
| Capsule biosynthesis and transport  (Campylobacter) | glf | orf02154 | 37 kDa outer-membrane protein mediating the binding of the organism to the extracellular matrix component fibronectin, which in turn stimulates a signal transduction pathway |
| Stj (Salmonella) | stjC | orf03398 | - |

**Table S2.** Antimicrobial resistance genes of H2 strain

| **Best_Hit_ARO** | **Best_Identities** | **Drug Class** | **Resistance Mechanism** | **AMR Gene Family** | **Percentage Length of Reference Sequence** |
| --- | --- | --- | --- | --- | --- |
| *acrA* | 99.75 | fluoroquinolone antibiotic; cephalosporin; glycylcycline; penam; tetracycline antibiotic; rifamycin antibiotic; phenicol antibiotic; disinfecting agents and antiseptics | antibiotic efflux | resistance-nodulation-cell division (RND) antibiotic efflux pump | 100 |
| *emrE* | 100 | macrolide antibiotic | antibiotic efflux | small multidrug resistance (SMR) antibiotic efflux pump | 100 |
| *kdpE* | 99.56 | aminoglycoside antibiotic | antibiotic efflux | kdpDE | 100 |
| *mdfA* | 96.59 | tetracycline antibiotic; disinfecting agents and antiseptics | antibiotic efflux | major facilitator superfamily (MFS) antibiotic efflux pump | 100 |
| *msbA* | 99.66 | nitroimidazole antibiotic | antibiotic efflux | ATP-binding cassette (ABC) antibiotic efflux pump | 100 |
| *mdtG* | 100 | phosphonic acid antibiotic | antibiotic efflux | major facilitator superfamily (MFS) antibiotic efflux pump | 100 |
| *mdtH* | 99.75 | fluoroquinolone antibiotic | antibiotic efflux | major facilitator superfamily (MFS) antibiotic efflux pump | 100 |
| *H-NS* | 100 | macrolide antibiotic; fluoroquinolone antibiotic; cephalosporin; cephamycin; penam; tetracycline antibiotic | antibiotic efflux | major facilitator superfamily (MFS) antibiotic efflux pump; resistance-nodulation-cell division (RND) antibiotic efflux pump | 100 |
| *marA* | 100 | fluoroquinolone antibiotic; monobactam; carbapenem; cephalosporin; glycylcycline; cephamycin; penam; tetracycline antibiotic; rifamycin antibiotic; phenicol antibiotic; penem; disinfecting agents and antiseptics | antibiotic efflux; reduced permeability to antibiotic | resistance-nodulation-cell division (RND) antibiotic efflux pump; General Bacterial Porin with reduced permeability to beta-lactams | 100 |
| *mdtA* | 98.8 | aminocoumarin antibiotic | antibiotic efflux | resistance-nodulation-cell division (RND) antibiotic efflux pump | 100 |
| *mdtB* | 99.71 | aminocoumarin antibiotic | antibiotic efflux | resistance-nodulation-cell division (RND) antibiotic efflux pump | 100 |
| *mdtC* | 99.71 | aminocoumarin antibiotic | antibiotic efflux | resistance-nodulation-cell division (RND) antibiotic efflux pump | 100 |
| *baeS* | 99.36 | aminoglycoside antibiotic; aminocoumarin antibiotic | antibiotic efflux | resistance-nodulation-cell division (RND) antibiotic efflux pump | 100 |
| *baeR* | 100 | aminoglycoside antibiotic; aminocoumarin antibiotic | antibiotic efflux | resistance-nodulation-cell division (RND) antibiotic efflux pump | 100 |
| *YojI* | 99.27 | peptide antibiotic | antibiotic efflux | ATP-binding cassette (ABC) antibiotic efflux pump | 100 |
| *PmrF* | 99.38 | peptide antibiotic | antibiotic target alteration | pmr phosphoethanolamine transferase | 100 |
| *emrY* | 99.61 | tetracycline antibiotic | antibiotic efflux | major facilitator superfamily (MFS) antibiotic efflux pump | 100 |
| *emrK* | 99.43 | tetracycline antibiotic | antibiotic efflux | major facilitator superfamily (MFS) antibiotic efflux pump | 110.26 |
| *evgA* | 100 | macrolide antibiotic; fluoroquinolone antibiotic; penam; tetracycline antibiotic | antibiotic efflux | major facilitator superfamily (MFS) antibiotic efflux pump; resistance-nodulation-cell division (RND) antibiotic efflux pump | 100 |
| *evgS* | 96.74 | macrolide antibiotic; fluoroquinolone antibiotic; penam; tetracycline antibiotic | antibiotic efflux | major facilitator superfamily (MFS) antibiotic efflux pump; resistance-nodulation-cell division (RND) antibiotic efflux pump | 100 |

**Table S3.** Antimicrobial resistance genes of plasmids found in H2 sample

| **Plasmid_ID** | **Best_Hit_ARO** | **Best_Identities** | **Drug Class** | **Resistance Mechanism** | **AMR Gene Family** | **Percentage Length of Reference Sequence** |
| --- | --- | --- | --- | --- | --- | --- |
| NZ_KR827684 | *tet(B)* | 99.25 | tetracycline antibiotic | antibiotic efflux | major facilitator superfamily (MFS) antibiotic efflux pump | 100 |
|  | *dfrA12* | 100 | diaminopyrimidine antibiotic | antibiotic target replacement | trimethoprim resistant dihydrofolate reductase (dfr) | 100 |
|  | *aadA2* | 100 | aminoglycoside antibiotic | antibiotic inactivation | ANT(3'') | 100 |
|  | *cmlA1* | 99.76 | phenicol antibiotic | antibiotic efflux | major facilitator superfamily (MFS) antibiotic efflux pump | 100 |
|  | *qacL* | 93.64 | disinfecting agents and antiseptics | antibiotic efflux | small multidrug resistance (SMR) antibiotic efflux pump | 100 |
|  | *sul3* | 100 | sulfonamide antibiotic | antibiotic target replacement | sulfonamide resistant sul | 100 |
| NZ_CP010149 | *sul2* | 100 | sulfonamide antibiotic | antibiotic target replacement | sulfonamide resistant sul | 100 |
|  | *APH(3'')-Ib* | 99.63 | aminoglycoside antibiotic | antibiotic inactivation | APH(3'') | 100 |
|  | *APH(6)-Id* | 99.28 | aminoglycoside antibiotic | antibiotic inactivation | APH(6) | 100 |
|  | *tet(A)* | 98.23 | tetracycline antibiotic | antibiotic efflux | major facilitator superfamily (MFS) antibiotic efflux pump | 94.1 |
|  | *CTX-M-14* | 100 | cephalosporin | antibiotic inactivation | CTX-M beta-lactamase | 100 |
|  | *TEM-1* | 100 | monobactam; cephalosporin; penam; penem | antibiotic inactivation | TEM beta-lactamase | 100 |
|  | *AAC(3)-IId* | 100 | aminoglycoside antibiotic | antibiotic inactivation | AAC(3) | 100 |
|  | *dfrA17* | 99.36 | diaminopyrimidine antibiotic | antibiotic target replacement | trimethoprim resistant dihydrofolate reductase dfr | 126.75 |
|  | *aadA5* | 100 | aminoglycoside antibiotic | antibiotic inactivation | ANT(3'') | 100 |
|  | *qacEdelta1* | 100 | disinfecting agents and antiseptics | antibiotic efflux | major facilitator superfamily (MFS) antibiotic efflux pump | 100 |
|  | *sul1* | 100 | sulfonamide antibiotic | antibiotic target replacement | sulfonamide resistant sul | 100 |
|  | *mphA* | 100 | macrolide antibiotic | antibiotic inactivation | macrolide phosphotransferase (MPH) | 100 |
|  | *sul2* | 100 | sulfonamide antibiotic | antibiotic target replacement | sulfonamide resistant sul | 100 |
|  | *APH(3'')-Ib* | 99.63 | aminoglycoside antibiotic | antibiotic inactivation | APH(3'') | 100 |
|  | *APH(6)-Id* | 99.28 | aminoglycoside antibiotic | antibiotic inactivation | APH(6) | 100 |
|  | *tet(A)* | 98.23 | tetracycline antibiotic | antibiotic efflux | major facilitator superfamily (MFS) antibiotic efflux pump | 94.1 |
|  | *sul3* | 100 | sulfonamide antibiotic | antibiotic target replacement | sulfonamide resistant sul | 100 |
|  | *qacL* | 93.64 | disinfecting agents and antiseptics | antibiotic efflux | small multidrug resistance (SMR) antibiotic efflux pump | 100 |
|  | *cmlA1* | 99.76 | phenicol antibiotic | antibiotic efflux | major facilitator superfamily (MFS) antibiotic efflux pump | 100 |
|  | *aadA2* | 100 | aminoglycoside antibiotic | antibiotic inactivation | ANT(3'') | 100 |
|  | *dfrA12* | 100 | diaminopyrimidine antibiotic | antibiotic target replacement | trimethoprim resistant dihydrofolate reductase dfr | 100 |
